# Different Circulation Pattern of Multiple Respiratory Viruses in Southern China During the COVID-19 Pandemic

**DOI:** 10.3389/fmicb.2021.801946

**Published:** 2022-01-26

**Authors:** Zhiqi Zeng, Wenda Guan, Yong Liu, Zhengshi Lin, Wenhua Liang, Jingyi Liang, Bingqian Chen, Tong Wu, Yutao Wang, Chunguang Yang, Qiubao Wu, Zhitong Mai, Jinchao Zhou, Junhou Zhou, Zhoulang Wang, Zhijie Lin, Chaohui Hu, Chunqiu Wu, Pengyuan Zhu, Canxiong Chen, Nanshan Zhong, Eric H. Y. Lau, Chitin Hon, Yaoming Liang, Zifeng Yang, Jianxing He

**Affiliations:** ^1^State Key Laboratory of Respiratory Disease, National Clinical Research Center for Respiratory Disease, Guangzhou Institute of Respiratory Health, The First Affiliated Hospital of Guangzhou Medical University, Guangzhou, China; ^2^Kingmed Virology Diagnostic and Translational Center, Guangzhou Kingmed Center for Clinical Laboratory Co., Ltd., Guangzhou, China; ^3^Singou Technology (Macau) Ltd., Macau, Macao SAR, China; ^4^Macao Institute of Systems Engineering, Macao University of Science and Technology, Macau, Macao SAR, China; ^5^Guangzhou KingCreate Biotechnology Co., Ltd., Guangzhou, China; ^6^School of Public Health, The University of Hong Kong, Hong Kong, Hong Kong SAR, China; ^7^Laboratory of Data Discovery for Health Limited (D^2^4H), Hong Kong Science Park, Hong Kong, Hong Kong SAR, China; ^8^Guangzhou KingMed Diagnostics Group Co., Ltd., Guangzhou, China; ^9^Guangzhou Laboratory, Guangzhou, China; ^10^Guangzhou Key Laboratory for Clinical Rapid Diagnosis and Early Warning of Infectious Diseases, Guangzhou Medical University, Guangzhou, China

**Keywords:** non-pharmaceutical interventions, SARS-CoV-2, respiratory viruses, influenza, circulation

## Abstract

China implemented stringent non-pharmaceutical interventions (NPIs) in spring 2020, which has effectively suppressed SARS-CoV-2. In this study, we utilized data from routine respiratory virus testing requests from physicians and examined circulation of 11 other respiratory viruses in Southern China, from January 1, 2018 to December 31, 2020. A total of 58,169 throat swabs from patients with acute respiratory tract infections (ARTIs) were collected and tested. We found that while the overall activity of respiratory viruses was lower during the period with stringent NPIs, virus activity rebounded shortly after the NPIs were relaxed and social activities resumed. Only influenza was effectively suppressed with very low circulation which extended to the end of 2020. Circulation of other respiratory viruses in the community was maintained even during the period of stringent interventions, especially for rhinovirus. Our study shows that NPIs against COVID-19 have different impacts on respiratory viruses.

## Introduction

As of November 2021, severe acute respiratory syndrome coronavirus 2 (SARS-CoV-2) has led to more than 250 million confirmed cases and 5 million deaths in the world (World Health Organization [WHO], 2021). Since the first case was reported in Wuhan, China in late 2019, the virus had quickly spread to all 31 provinces across China by January 2020. Guangdong province is located in the southern part of mainland China, with a land area of 179,800 square kilometers and a population of more than 100 million. Guangdong identified the first COVID-19 case on January 19, 2020, 4 days prior to the lockdown in Wuhan. The Guangdong government took immediate actions to control the epidemic, including restriction of public gatherings and suspension of school and work. Wearing facemasks and keeping physical distance in public venues were made mandatory (Yang et al., 2020). Within 2 months, the COVID-19 outbreak was effectively put under control in the province. The focus of interventions then shifted toward control of local spread from imported cases, while wearing facemasks in public venues was still encouraged (People’s Government of Guangdong Province, 2020a,b). As the local epidemic improved, social activities, schools, and businesses have gradually resumed to pre-epidemic level in the second half of 2020. During this period, no local COVID-19 case has been reported in Guangdong.

SARS-CoV-2 infection can spread through aerosols and respiratory droplets (Jayaweera et al., 2020). Non-pharmaceutical interventions (NPIs) such as social distancing, wearing facemasks and eye protective gears, are effective to reduce person-to-person transmission of SARS-CoV-2 (Chu et al., 2020). As other respiratory viruses, such as influenza, spread via the same transmission routes, some transmission of these viruses should also be prevented by these measures. Indeed, a study in Guangzhou, the capital city of Guangdong province, showed that influenza activity dropped dramatically since the first few months of 2020 when control measures were implemented (Wu et al., 2020). In this study, we examined the effect of NPIs on other respiratory viruses, and also circulation of different respiratory viruses when NPIs were relaxed.

## Materials and Methods

### Sample Collection

From January 2018 to December 2020, a total of 58,169 patients with acute respiratory tract infections (ARTIs) were enrolled from multiple regions across Guangdong province. Inclusion criteria were having a fever (≥37.3°C), accompanied by at least one of the respiratory symptoms, such as cough, sore throat, coryza, or shortness of breath. All patients satisfying the inclusion criteria with a laboratory test request from their physicians were included. Throat swab samples were refrigerated at 2–8°C in viral transport medium and transported to the laboratory of Kingmed Diagnostics (KMD) which were either immediately tested or stored at –80°C until further testing. KMD^1^ is an accredited commercial medical laboratory in China (Luo et al., 2019). The central laboratory of KMD is located in Guangzhou, which provides viral testing services to 21 cities and 262 hospitals in Guangdong province. Most of these patients were pediatric outpatients and hence our samples reflected virus activity among those with mild respiratory symptoms. From January 2020 onward, all patients with fever or suspected with COVID-19 were first screened by SARS-CoV-2 nucleic acid test in the clinics or hospitals, and only samples from those who were tested negative were sent to KMD for testing of other respiratory viruses. COVID-19 case counts were collected from Chinese Center for Disease Control and Prevention (2020). Mobility data was collected from National Bureau of Statistics of China (2020) which is a combined measure of local mobility by railway and other public transports, from China State Railway Group Co., Ltd. And Ministry of Transport of the People’s Republic of China, respectively.

### Virological Testing

Sample testing for respiratory viruses were requested by physicians to assist diagnosis and patient management. Throat swab samples were tested using TaqMan real-time PCR assays. Viral RNA/DNA was extracted using the Nucleic acid extraction kit (Shanghai Kehua Bio-Engineering Co., Ltd.). Influenza A (IAV) and influenza B virus (IBV) were tested using the TaqMan real-time PCR testing kit (ZJ Bio-Tech, Shanghai, China). Human metapneumovirus (HMPV), parainfluenza virus (PIV), adenovirus (ADV), rhinovirus (RHV), respiratory syncytial virus (RSV), human coronaviruses (HCoV)-OC43, HCoV-HKU1, HCoV-NL63, and HCoV-229E were identified using the TaqMan real-time PCR testing kit (Hecin-Scientific, Guangzhou, China). All procedures were performed according to the manufacturers’ protocol. Testing service was expanded to cover HMPV and RSV in May and June 2018, respectively.

### Statistical Analysis

We used descriptive statistics to summarize epidemiological characteristics of the respiratory viruses. We also tested the difference in the detection rate of each respiratory virus in specific periods with stringent control measures and relaxation in 2020, vs. the same periods in 2018-19 using Chi-Square test. Statistical significance was defined as *P* < 0.05. Data were analyzed using Python (version 3.7).

## Results

**FIGURE 1:**
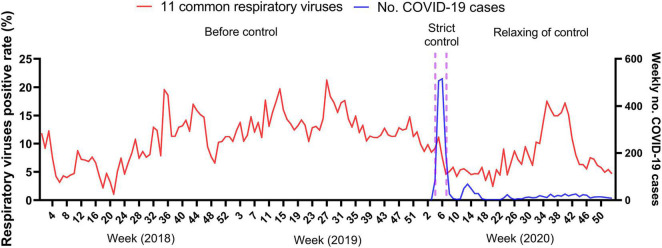
Weekly detection of respiratory viruses from January 2018 to December 2020. Incidence of 11 common respiratory viruses and weekly number of confirmed COVID-19 cases. Positive rate (%) was calculated as the weekly number of positive detections of 11 common respiratory viruses divided by the weekly total number of tests for individual respiratory viruses. Detection of respiratory viruses was lower before June 2018 (week 21 of 2018), as testing for HMPV and RSV started in May and June 2018, respectively.

We analyzed 11 respiratory viruses detected from 58,169 ARTI patients from samples collected before, during and after the local COVID-19 epidemic. The median age of the patients was 3.0 years (interquartile range, 1.0–6.6 years), and the male to female ratio was 1.5:1. 23.4% of these patients were hospitalized (Table 1). The Guangdong provincial government implemented stringent control measures against COVID-19 in late January (week 4, 2020), soon after reporting of the first local cases. The series of control measures (Supplementary Figure 1) have successfully suppressed local COVID-19 cases (Supplementary Figure 2). Just before the public interventions were implemented, the overall weekly detection rate of 11 respiratory viruses that we studied was about 10% in the first 3 weeks of 2020, following a gentle decline since mid-2019 (Figure 1). Both the respiratory virus detection rate and the number of COVID-19 cases decreased sharply at week 7 after stringent measures were implemented, particularly for COVID-19 cases. As the public interventions started to relax gradually beginning at week 8 of 2020, respiratory virus activity remained at a low level (< 10%) for the following 15 weeks until week 22, while there was a mild resurgence of COVID-19 cases in week 12–16. Thereafter, the respiratory virus detection rate increased gradually and peaked at 17% in week 35. There were large variations in the positive rates across the 11 respiratory viruses.

**TABLE 1:** Demographic characteristics of all enrolled patients with ARTI, 2018 to 2020.

	2018 (*N* = 7,611)		2019 (*N* = 31,764)		2020 (*N* = 18,794)		Overall (*N* = 58,169)	
	*n*	%	*n*	%	*n*	%	*n*	%
**Sex**								
Male	4,557	59.9	18,034	56.8	11,084	59.0	33,675	57.9
Female	2,948	38.7	12,652	39.8	7,373	39.2	22,973	39.5
Missing	106	1.4	1,078	3.4	337	1.8	1,521	2.6
**Age groups (y)**								
0–4	5,677	74.6	21,231	66.9	12,926	68.8	39,834	68.5
5–18	1,068	14.0	6,828	21.5	2,147	11.4	10,043	17.3
19–49	449	5.9	1,541	4.9	1,024	5.5	3,014	5.2
50–64	183	2.4	604	1.9	561	3.0	1,348	2.3
≥ 65	195	2.6	1,108	3.5	759	4.0	2,062	3.5
Missing	39	0.5	452	1.4	1,377	7.3	1,868	3.2
**Settings**								
Inpatients	590	7.8	9,511	29.9	3,515	18.7	13,616	23.4
Outpatients	4,878	64.1	18,490	58.2	5,192	27.6	28,560	49.1
Missing	2,143	28.2	3,763	11.9	10,087	53.7	15,993	27.5

We found that 11,878 of the 58,169 (20.4%) patients were tested positive in 2018–2020 for any of the 11 respiratory viruses. RSV (15.1%, 1,926 positive cases/12,725 tests) and ADV (14.7%, 5,829 positive cases/39,543 tests) were most prevalent, followed by RHV, PIV, IAV, and IBV.

### Circulation of Respiratory Viruses Before, During and After the Local COVID-19 Epidemic

**FIGURE 2:**
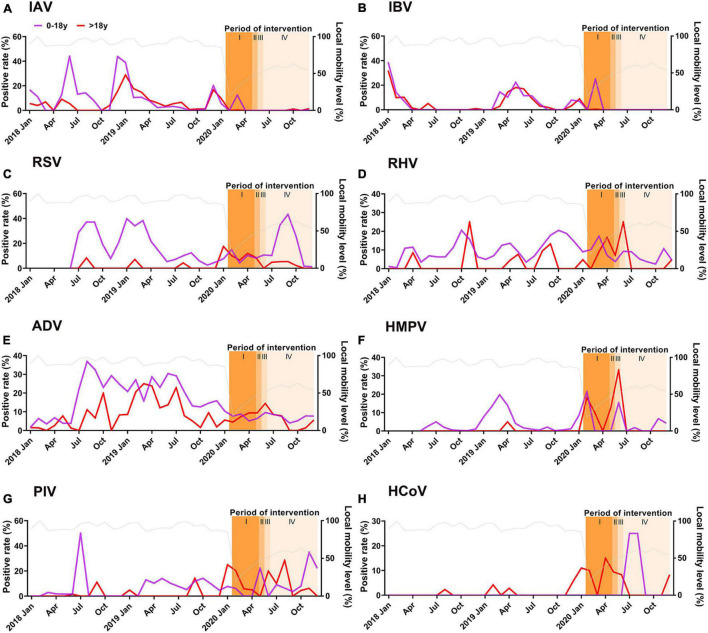
Monthly detection of 8 respiratory viruses, 2018–2020, including periods before, during and after the local COVID-19 epidemic and after workplace and school reopening. **(A)** IAV **(B)** IBV **(C)** RSV (June 2018–December 2020) **(D)** RHV **(E)** ADV **(F)** HMPV (May 2018-December 2020) **(G)** PIV **(H)** HCoV including HCoV-OC43, HCoV-HKU1, HCoV-NL63, and HCoV-229 **(E)**. Positive rate: percentage of positive test results divided by the number of tests for each respiratory virus. Local mobility level (%) (Gray line): the passenger flow of roads and railways in different months divided by the maximum passenger flow of road and railways recorded in February 2018. Shaded bars “I” indicated the period with the most stringent control measures and school closure. Shaded bars “II” indicated the period when control measures were relaxed gradually and first phase of school reopening (all senior and junior high school students). Shaded bars “III” indicated the second phase of school opening (extended to all elementary and college students). Shaded bars “IV” indicated full resumption of all schools (extended to kindergarteners).

We analyzed the activity of 8 respiratory viruses separately during 2018–2020, including the period with the most stringent control measures against COVID-19 (February to May, 2020) and the following period where interventions were relaxed and social activities resumed (Figure 2). Mobility dropped to a very low level only for the first few weeks of February, and then rebounded afterward though it has not returned to the pre-pandemic level by the end of 2020 (Figure 2). Test volume for most respiratory viruses reduced significantly during February to May 2020 due to the urgent deployment of laboratory resources for SARS-CoV-2 testing, and gradually resumed to normal level afterward (Supplementary Figure 3). Activity of RSV among children and HCoV for all ages exceeded those before the COVID-19 epidemic.

Influenza virus usually had higher activity in winter (December to February in Guangzhou) and spring (March to May) before the COVID-19 pandemic (Figures 2A,B). However, influenza activity in early 2020 was interrupted quickly after implementation of the NPIs. RSV and HMPV also showed clear peaks in 2019 spring, and RSV had elevated activity in late summer. Though data for RSV and HMPV were not available in early 2018, other studies have reported spring peaks in Southern China in previous years (Zhang et al., 2014; Liao et al., 2015). The spring peaks for both viruses were suppressed in 2020 when NPIs were implemented. Typically, there is high RHV activity in spring and autumn, matching with the usual school periods. Overall, IAV, IBV, RSV, and ADV activities were reduced significantly during the period with most stringent interventions (Table 2).

**TABLE 2:** Detection rates of respiratory viruses during the period with most stringent control measure against COVID-19 in 2020 (period I) and the following period when the measures were relaxed (period II-IV).

Virus	Detection rate (%)					
	Period I^a^			Period II-IV^b^		
	2018–19	2020	*P*-value^c^	2018–19	2020	*P*-value*^d^*
IAV	13.0	2.5	<0.001	7.8	0.0	<0.001
IBV	7.4	2.2	<0.001	7.2	0.0	<0.001
RSV	28.8	10.0	<0.001	8.3	21.6	<0.001
RHV	9.7	9.2	0.800	11.4	7.2	<0.001
ADV	18.6	8.3	<0.001	19.0	8.1	<0.001
HMPV	14.9	10.1	0.244	1.6	4.6	0.022
PIV	3.9	11.9	<0.001	8.4	8.8	0.958
HCoVs	1.3	10.5	<0.001	1.1	11.8	<0.001

*Detection rates in the same period in 2018–2019 were also present and compared.*

*^a^January 20 to April 26.*

*^b^April 27 to December 31.*

*^c^Comparison of respiratory virus detection rates between period I, 2018–2019 vs. period I, 2020 using Chi-Square test.*

*^d^Comparison of respiratory virus detection rates between period II-IV, 2018–2019 vs. period II-IV, 2020 using Chi-Square test.*

Interestingly, circulation and seasonality of RHV seemed to have maintained over the COVID-19 epidemic (Figure 2). ADV activity showed less prominent seasonality and less circulated over the years, and the impact from NPIs was uncertain. We also observed clear age differences. Circulation of RSV, RHV, and HMPV in younger population was much higher, but there was no clear difference in the seasonality between children and adults.

Social activities including schools and work resumed gradually in May, and all schools including kindergartens reopened in early June. However, influenza activity remained at an extremely low level even to the end of 2020 (Table 2). In contrast, RSV resumed its typical peak in late summer. RHV activity was not much affected by the control measures in both children and adults. Similarly, ADV in children remained at a low level after schools had resumed. The patterns of PIV and HCoVs were less clear as limited samples were collected since the COVID-19 pandemic.

### Co-infections in Acute Respiratory Tract Infections Patients

We also detected co-infections in ARTI patients, though for each patient only some of the 11 pathogens were tested according to physician’s request. Of the 58,169 patients, 368 (0.006%) were infected with more than one respiratory pathogens (Supplementary Table 1). Of these, ADV/RHV (35.1%, 129/368) co-infection was most frequent, followed by RSV/ADV (23.4%, 86/368), ADV/PIV (9.0%, 33/368) and RSV/RHV (8.2%, 30/368).

## Discussion

In this study, we examined the epidemiological characteristics of common respiratory viruses that caused ARTI infections in Guangzhou, Guangdong province, China, and compared their activity before, during and after the local COVID-19 epidemic. Our study analyzed a large number of samples from > 50,000 ARTI patients, collected from multiple sites over 3 years. In the first wave of COVID-19 epidemic in Guangdong, mainly driven by the imported cases from Wuhan and local transmission, stringent measures have been implemented immediately. Such measures have then shifted toward control against spillover from imported cases and travel restriction, while local NPIs relaxed when local epidemics died out. Along with refinement of local control strategy with lockdown of neighborhoods when cases were identified, this strategy has allowed Guangdong to maintain low case counts in the second half of the year. We described the activity of common respiratory viruses in children and adults in this unique epidemiological situation when most of the social activities resumed after the local COVID-19 epidemics. We observed that influenza viruses have been effectively suppressed through the end of 2020.

We found that RSV and ADV were the most commonly detected respiratory viruses in 2018–2020, followed by RHV, PIV and influenza virus A and B. This finding is similar to an earlier study in 2009–2014 in Southern China where influenza, RSV and ADV were found to be the most common respiratory viruses (Zhang et al., 2014; Liao et al., 2015). Most of our samples were collected from outpatients and hence better reflected respiratory virus activity in the community, when compared to studies which focused on hospitalized patients and may be affected by revised admission criteria as part of the pandemic response (Fukuda et al., 2021; Liu et al., 2021). In early 2020 when the most stringent control measures against COVID-19 were implemented, influenza viruses among children and adults were suppressed to a historically low level in Guangzhou as well as in many parts of the world (Olsen et al., 2020; Zipfel et al., 2021). As the number of local COVID-19 cases in Guangzhou dropped quickly, social activity resumes relatively quickly to pre-pandemic level by mid-April 2020 (Huang et al., 2021), though with reduced mobility from the pre-pandemic level (Figure 2). This may suggest a change in social contact pattern but not overall volume. The very low influenza activity was sustained in Guangzhou even after relaxation of the NPIs, while some tropical Asian countries already reported detection of influenza viruses in the second half of 2020 (Mott et al., 2021). The stringent travel restrictions on inbound travels in mainland China have probably delayed the return of influenza virus in the community.

Meanwhile, the impact of NPIs on some other respiratory viruses was more limited in Guangzhou. Such phenomenon was also reported in Australia, Finland, Italy, Japan, the US and elsewhere (Sberna et al., 2020; Sullivan et al., 2020; Kuitunen et al., 2021; Takashita et al., 2021). In particular, RHV was also reported to circulate as usual among children in Finland (Kuitunen et al., 2021) or even higher than usual in Japan and Australia (Sullivan et al., 2020; Takashita et al., 2021), during the period with various control measures against COVID-19. In Guangzhou, we observed weaker suppression of non-enveloped viruses (RHV, ADV in our study) compared to enveloped viruses (influenza, RSV and HMPV in our study), as have also been reported in South Korea (Park et al., 2021). Although mandatory mass wearing of facemasks in public space, along with other control measures, was strictly enforced in the first few months of the COVID-19 epidemic, we only observed strong effects on COVID-19 and influenza. This could be partly explained by surgical facemasks having a lower efficiency in preventing transmission of rhinovirus compared to influenza and coronavirus (Leung et al., 2020). While it is known that the transmission routes of these viruses were mainly airborne and direct contact, the contribution of each route by each virus is not clear. The NPIs may have different effect on these transmission routes partly explaining the observed differences. Further studies are needed to understand the observed differences in the impact of NPIs on different respiratory viruses.

We observed changes in seasonal pattern of some respiratory viruses due to the COVID-19 epidemic. The stringent NPIs have almost eliminated the spring-summer peaks for influenza, while the late summer peak of RSV reappeared as usual when the control measures were relaxed, especially among children. After schools reopened, we found that respiratory viruses spread more quickly among children, especially for RSV. In Austria, RSV and HMPV activity remained very low after relaxation of restriction at a period coinciding with the typical off-season in summer, meanwhile RHV detection rebounded quickly (Redlberger-Fritz et al., 2021). In Guangdong and elsewhere, RHV remained its usual circulation level even during the period with the most stringent NPIs and school closure; hence, it is not indicative of the within-school transmission potential of other respiratory viruses during COVID-19. The exceptionally low circulation of influenza for more than 1 year implies that the general population was not exposed to influenza virus from some time, resulting in lower population immunity. Some of the preventive measures against COVID-19 should be encouraged when influenza returns, to avoid overloading of the healthcare capacity.

There are some limitations in the study. First, submission of test requests for respiratory viruses other than COVID-19 dropped dramatically during the early COVID-19 epidemic due to public health emergence, resulting in more unstable detection rates. Second, tests for a specific virus instead of multiple viruses were requested by physicians to aid diagnosis. Test requests for ADV, RHV, RSV were most frequent, but usually only 1–2 viruses were requested for each patient. Third, adults may have milder clinical presentation for some respiratory viruses and are less likely to seek for medical attention. Finally, testing for HMPV and RSV started in May and June 2018, respectively. Results from other studies in the region were used to determine their seasonality.

## Conclusion

Stringent public health interventions against COVID-19 in Southern China have effectively suppressed both SARS-CoV-2 and influenza circulation; however, its impact on other common respiratory viruses was much more limited. Routine respiratory virus testing requests from physicians provided an opportunity to monitor virus circulation in the community. As mass COVID-19 vaccination has been rolled out in many places worldwide and provides the basis for further relaxation of the control measures, circulation of respiratory viruses such as RHV, RSV, and ADV are expected to return to pre-pandemic level. Understanding the differential effectiveness of public health measures on different pathogens would improve early preparedness.

## Data Availability Statement

The datasets generated for this study are available on request to the corresponding author.

## Ethics Statement

This study only included anonymized surveillance data without personal identifiers, thus no ethical approval is needed. This study did not affect the diagnosis or the therapeutic strategy.

## Author Contributions

JH, ZY, YML, CH, and EL contributed to conceptualization, ZZ, WG, YL, ZSL, WL, JL, and EL contributed to draft the manuscript. BC, YW, CY, QW, ZM, JCZ, JHZ, ZW, ZJL, and CC contributed to collect data and produced outputs, CHH, CW, and PZ contributed to laboratory analysis, JH, ZY, YML, and NZ contributed to supervision, ZZ, ZSL, and EL contributed to review and editing. All authors read and approved the final manuscript.

## Conflict of Interest

YL was employed by company Guangzhou Kingmed Center for Clinical Laboratory Co., Ltd. TW was employed by company Singou Technology (Macau) Ltd. CHH, CW, and PZ were employed by company Guangzhou KingCreate Biotechnology Co., Ltd. YML was employed by company Guangzhou KingMed Diagnostics Group Co., Ltd. The remaining authors declare that the research was conducted in the absence of any commercial or financial relationships that could be construed as a potential conflict of interest.

## Publisher’s Note

All claims expressed in this article are solely those of the authors and do not necessarily represent those of their affiliated organizations, or those of the publisher, the editors and the reviewers. Any product that may be evaluated in this article, or claim that may be made by its manufacturer, is not guaranteed or endorsed by the publisher.

## Footnotes

1
http://en.kingmed.com.cn
