# Supplementary material for: Different Circulation Pattern of Multiple Respiratory Viruses in Southern China During the COVID-19 Pandemic
Source: Front Microbiol. 2022 Jan 26;12:801946. doi: 10.3389/fmicb.2021.801946 (PMC8826816; doi:10.3389/fmicb.2021.801946)
Supplement: Supplementary file 1 [file Data_Sheet_1.docx]

**Supplemental material**


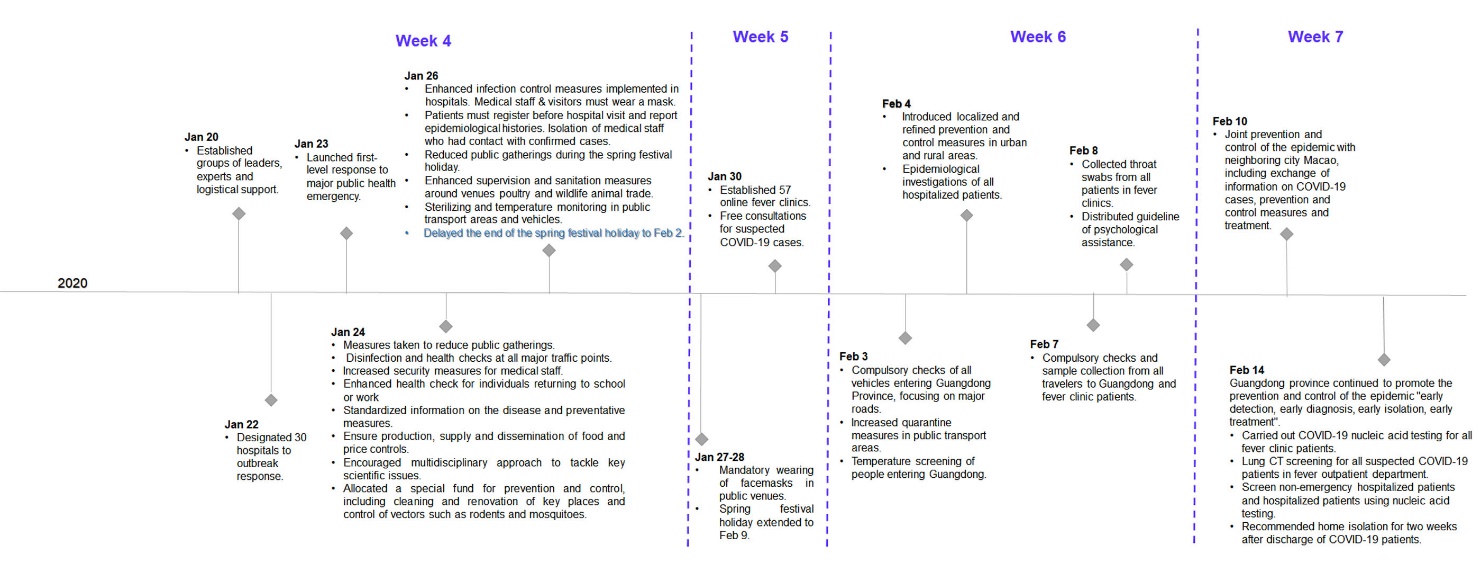


**Figure S1. Summary of strict control measures introduced in Guangdong.** The control measures for COVID-19 were referred from Yang et al., 2020 and collected from the official website of the People's Government of Guangdong Province (http://www.gd.gov.cn/gdywdt/zwzt/yqfk/gdzxd/).


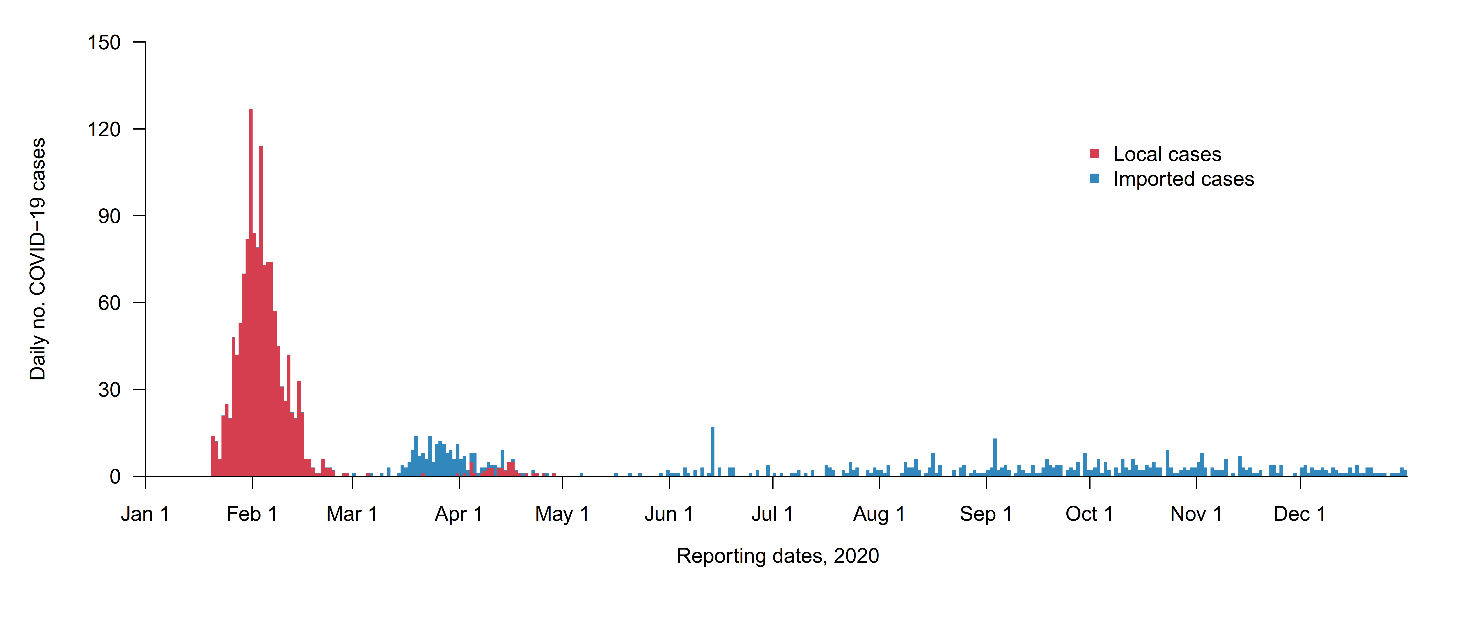


**Figure S2. Number of local and imported COVID-19 cases, 2020.** The local cases and imported cases of COVID-19 were collected from the official website of the Health Commission of Guangdong Province (http://wsjkw.gd.gov.cn/).


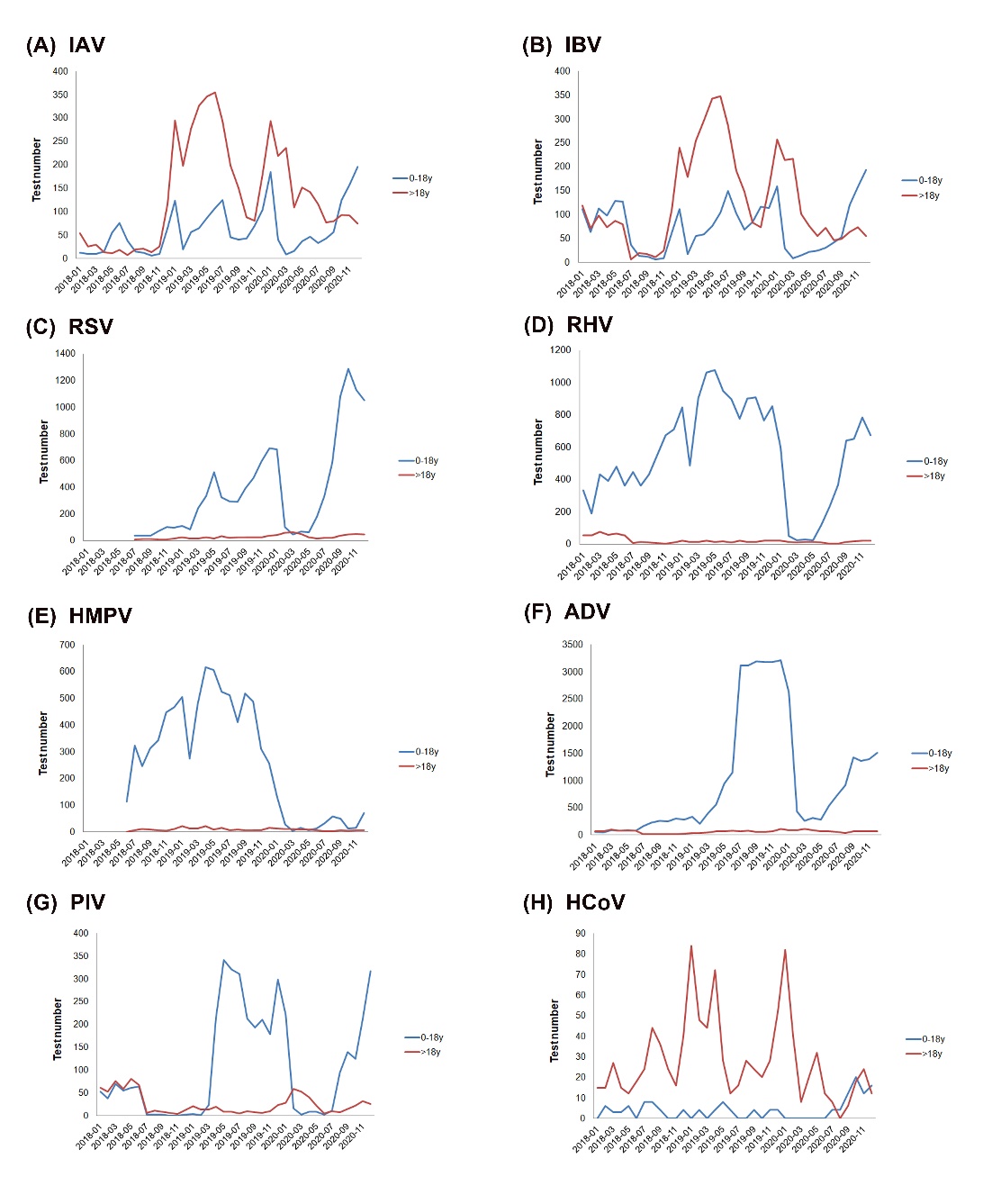


**Figure S3. Monthly number of tests for individual respiratory viruses**

**Table S1. Co-pathogen detection in ARTI patients**

| Two Pathogens | n | % |  | n | % |
| --- | --- | --- | --- | --- | --- |
| ADV, RHV | 129 | 0.22 | ADV, IBV | 2 | 0.003 |
| ADV, RSV | 86 | 0.15 | HCoV-NL63, HMPV | 1 | 0.002 |
| ADV, PIV | 33 | 0.06 | HCoV-NL63, RSV | 1 | 0.002 |
| RHV, RSV | 30 | 0.05 | HCoV-HKU1, ADV | 1 | 0.002 |
| RHV, PIV | 19 | 0.03 | HCoV-HKU1, PIV | 1 | 0.002 |
| RHV, HMPV | 17 | 0.03 | HMPV, PIV | 1 | 0.002 |
| RSV, PIV | 15 | 0.03 | HMPV, IBV | 1 | 0.002 |
| IAV, IBV | 8 | 0.01 | HCoV-OC43, ADV | 1 | 0.002 |
| IAV, ADV | 6 | 0.01 | HCoV-OC43, PIV | 1 | 0.002 |
| RSV, HMPV | 2 | 0.003 | RSV, IAV | 1 | 0.002 |
|  | | | | | |
| Three Pathogens |  | |  |  |  |
| RHV, PIV, ADV | 4 | 0.007 | RSV, HCoV-NL63, RHV | 1 | 0.002 |
| RHV, PIV, RSV | 3 | 0.005 | HCoV-OC43, HMPV, HCoV-NL63 | 1 | 0.002 |
| ADV, HCoV-229E, HCoV-HKU1 | 1 | 0.002 | HCoV-OC43, HMPV, RHV | 1 | 0.002 |
| RSV, ADV, RHV | 1 | 0.002 |  |  |  |

**REFERENCES**

Yang, Z., Zeng, Z., Wang, K., Wong, S.S., Liang, W., Zanin, M., et al. (2020). Modified SEIR and AI prediction of the epidemics trend of COVID-19 in China under public health interventions. J Thorac Dis 12(3), 165-174. doi: 10.21037/jtd.2020.02.64.
